# The effect of mild-to-moderate hearing loss on auditory and emotion processing networks

**DOI:** 10.3389/fnsys.2014.00010

**Published:** 2014-02-04

**Authors:** Fatima T. Husain, Jake R. Carpenter-Thompson, Sara A. Schmidt

**Affiliations:** ^1^Department of Speech and Hearing Science, University of Illinois at Urbana-ChampaignChampaign, IL, USA; ^2^The Neuroscience Program, University of Illinois at Urbana-ChampaignChampaign, IL, USA; ^3^Beckman Institute for Advanced Science and Technology, University of Illinois at Urbana-ChampaignChampaign, IL, USA

**Keywords:** fMRI, hearing loss, resting-state fMRI, functional connectivity, emotion, IADS

## Abstract

We investigated the impact of hearing loss (HL) on emotional processing using task- and rest-based functional magnetic resonance imaging. Two age-matched groups of middle-aged participants were recruited: one with bilateral high-frequency HL and a control group with normal hearing (NH). During the task-based portion of the experiment, participants were instructed to rate affective stimuli from the International Affective Digital Sounds (IADS) database as pleasant, unpleasant, or neutral. In the resting state experiment, participants were told to fixate on a “+” sign on a screen for 5 min. The results of both the task-based and resting state studies suggest that NH and HL patients differ in their emotional response. Specifically, in the task-based study, we found slower response to affective but not neutral sounds by the HL group compared to the NH group. This was reflected in the brain activation patterns, with the NH group employing the expected limbic and auditory regions including the left amygdala, left parahippocampus, right middle temporal gyrus and left superior temporal gyrus to a greater extent in processing affective stimuli when compared to the HL group. In the resting state study, we observed no significant differences in connectivity of the auditory network between the groups. In the dorsal attention network (DAN), HL patients exhibited decreased connectivity between seed regions and left insula and left postcentral gyrus compared to controls. The default mode network (DMN) was also altered, showing increased connectivity between seeds and left middle frontal gyrus in the HL group. Further targeted analysis revealed increased intrinsic connectivity between the right middle temporal gyrus and the right precentral gyrus. The results from both studies suggest neuronal reorganization as a consequence of HL, most notably in networks responding to emotional sounds.

## Introduction

Hearing loss (HL) remains one of the most common chronic conditions affecting older adults (Cruickshanks et al., 1998), with the prevalence rate increasing from between 25 and 40% in adults above 65 years of age to greater than 80% in people older than 85 years (Yueh et al., 2003). In general, mild-to-moderately severe sensorineural HL, which is often untreated, affects about 23–33% of the adult population in the world (Stevens et al., 2013). HL has a significant impact on quality of life and general well-being of an older adult (Mulrow et al., 1990; Carabellese et al., 1993) and may be associated with depression and isolation, especially in those younger than 70 years of age (Gopinath et al., 2009). However, little is known about the consequences of mild-to-moderately severe HL on the neural architecture and functionality of the brain.

The majority of brain imaging studies that have explored HL have done so when HL has occurred in conjunction with tinnitus (e.g., Weisz et al., 2004; Husain et al., 2011b), other disorders (e.g., Yoneda et al., 2012) or in the context of sign language studies when the impairment has been profound (e.g., Petitto et al., 2000; Husain et al., 2009). A few brain imaging studies have investigated the impact of HL on aging (Wong et al., 2010; Peelle et al., 2011); these remain the best sources to understand the neural correlates of HL. These neural correlates are manifested in decrease in gray matter in the frontal cortex (Wong et al., 2010; Peelle et al., 2011) and a decreased response in the superior temporal cortex, thalamus and brainstem in a speech comprehension task (Peelle et al., 2011). Our previous structural MRI study of HL in older adults (conducted as part of a larger study to investigate neural bases of tinnitus and HL) observed gray matter loss in the frontal cortices and disordered white matter tracts leading into and out of the auditory cortex (Husain et al., 2011a). A companion functional study noted increased response of the regions in the frontal and parietal cortices, possibly which related to increased attention processing (Husain et al., 2011b). In the latter fMRI study, participants had mild-to-moderately severe HL with an average age in the mid-fifties and were asked to perform a discrimination task with simple tones and tonal sweeps. When compared to rest trials, task trials resulted in greater response in the temporal, frontal and parietal cortices in the HL group relative to the normal hearing (NH) controls.

In the present study, we investigated the neural correlates of mild-to-moderate sensorineural HL in middle-aged adults without tinnitus or any other chronic physical or mental condition and compared them to age-matched NH controls. We concentrated on extra-auditory networks, specifically the one concerned with emotional processing. The limbic system is typically the main network of emotion processing. It consists primarily of the amygdala, parahippocampus, ventral medial prefrontal cortex, nucleus accumbens, and insula. Recently, studies have begun to map out the regions and connectivity of the auditory emotional network in adults with NH (Blood and Zatorre, 2001; Koelsch et al., 2006; Kumar et al., 2012) and in those with tinnitus (Giraud et al., 1999; Mirz et al., 2000; Seydell-Greenwald et al., 2012; Golm et al., 2013). Using dynamic causal modeling, Kumar et al. (2012) found that the negative valence of a sound modulates the backward connections from the amygdala to the auditory cortex, and the acoustic features of a sound modulate the forward connections from the auditory cortex to the amygdala. It is likely that such acoustic features, processing nodes and their connectivity may be susceptible to changes due to sustained loss of hearing acuity when listening to affective sounds. This may result in delayed processing or misclassification or both of the affective sounds. One of the goals of the present study was to investigate whether loss of hearing acuity affects the acoustic processing of affective sounds and whether this impacts their perception.

HL may also affect the perception of the valence of affective sounds, regardless of the processing of acoustic features. Tinnitus, in particular, has been established to have an altered auditory-limbic system link (Jastreboff, 1990; Rauschecker et al., 2010); behaviorally, there is greater prevalence of depression and anxiety in the tinnitus patient group compared to the general population (Bartels et al., 2008). Not surprisingly then, this disordered link is beginning to be studied in tinnitus. However, HL occurs in about 90% of the individuals with tinnitus (Davis and Rafaie, 2000), and the unique contribution of tinnitus to any changes in emotional processing is unknown. There are other reasons to study emotional processing in HL. As previously noted, prevalence of HL increases with age (Yueh et al., 2003) and may contribute to social isolation (Gopinath et al., 2009). This in turn may impact the emotion processing limbic network, as it has been shown to occur with aging and with tinnitus (Mather and Knight, 2005; Rauschecker et al., 2010; St Jacques et al., 2010; Anticevic et al., 2012). Nevertheless, no brain imaging study has explicitly investigated the emotion processing network in older adults with HL.

We conducted both a task-based and a resting state functional connectivity study of the emotion processing network in middle-aged adults with bilateral sensorineural HL. The task consisted of classification of sounds as being “pleasant,” “unpleasant,” or “neutral.” Our working hypothesis was that a loss of hearing acuity affects behavior, sounds may appear more unpleasant (Franks, 1982; Feldmann and Kumpf, 1988; Leek et al., 2008; Rutledge, 2009; Uys et al., 2012), reaction times may be longer due to effortful listening (Hicks and Tharpe, 2002; Tun et al., 2009). Likewise, the neural network subserving emotion processing may be affected, specifically in the response patterns of the nodes. In order to assess the impact of HL on a baseline, resting state, without the distraction of any task, we measured the functional connectivity of a number of networks, including that connected to the amygdala (a primary node of the limbic system).

Our main focus was on auditory and limbic systems, but these systems interact with intrinsic networks devoted to attention processing and possibly the default mode network (DMN). Intrinsic networks, or resting state networks, are defined as spontaneous, low-frequency oscillations in brain activity that can be organized into coherent networks. In many cases, intrinsic networks mirror task-related networks. For example, the auditory resting state network closely resembles an auditory task network. However, instead of correlations between activated regions in the task-based network, the intrinsic network shows correlations between deactivated brain regions. The DMN is the quintessential resting state network and is unique in that it exhibits deactivation in a task-based state and is active during rest (Raichle et al., 2001). The DMN exhibits a push-pull type of relationship with other networks in the brain (Fox et al., 2005). The dorsal attention network (DAN), for instance, will show activations while the DMN is deactivated (in a task-based state). Its relationship with the DAN and other intrinsic networks warrants study of the DMN. Many disorders, including Alzheimer's disease, schizophrenia, and tinnitus, have been shown to affect the connectivity of the DMN (for reviews see, Greicius, 2008; Husain and Schmidt, 2013). It is also possible that intrinsic connectivity may differ in patients with HL, perhaps relating in particular to limbic areas. Alterations to resting state functional connectivity may result in decreased preparedness to perform a task. In particular, if limbic areas are shown to display irregular connectivity to other brain regions at rest, it may change how individuals process emotional stimuli.

Although we had provisional hypotheses, our study was exploratory because of a lack of brain imaging studies investigating the impact of HL on emotion processing and related extra-auditory networks. In the resting state portion of our study, general hypotheses were made regarding which networks may show altered connectivity, but we did not specify the nodes and the nature of these alterations. We had more constrained expectations about the role of amygdala and auditory processing areas in the emotion-task study, in that we expected reduced engagement of such regions in listeners with HL when processing affective stimuli. In sum, we conducted a comprehensive study, combining both task- and rest-based fMRI using multiple seed regions in order to establish a baseline for future studies.

## Methods

### Subjects

Participants were recruited from the Urbana-Champaign area, were scanned under the UIUC IRB 10144 protocol, gave written informed consent, and were monetarily compensated. Subjects belonged to one of two groups: middle-aged adults with bilateral high-frequency sensorineural HL (*n* = 12), or their age- and gender-matched controls with NH (*n* = 15). Three NH subjects were excluded due to excessive motion artifact, and data from only 12 NH participants were included in the final analysis. During recruitment, we excluded subjects that presented with tinnitus or with asymmetric HL at the time of their audiological examination. We defined asymmetric HL to be more than a 15 dB HL difference between the right and left ear at one or more frequencies, or if the right and left ear differed by 10dB HL at two consecutive frequencies. The Beck Depression Inventory (BDI-II) and the Beck Anxiety Inventory (BAI) were used to assess depression and anxiety levels (Beck and Steer, 1984; Steer et al., 1993, 1999). All subjects scored in the minimal depression range and minimal anxiety range for the BDI-II and BAI, respectively. See Table 1 for information about subject demographics.

**Table 1:** **Subject demographics and clinical characteristics for the subject groups**.

**Group**	**NH (Normal hearing)**		**HL (Hearing loss)**	
Group size	12		12	
Age(M ± *SD*)	51.4 ± 9.9		58.2 ± 9.5	
Gender	6 males, 6 females		5 males, 7 females	
BAI(M ± *SD*)	1.25 ± 1.3		2.3 ± 1.7	
BDI-II(M ± *SD*)	1.7 ± 2.3		4.3 ± 4.1	
Average hearing threshold (dB HL, right column) at different testing frequencies (left column) (M ± *SD*)	0.5 kHz	13.5 ± 8.8	0.5 kHz	15.0 ± 8.7
	1 kHz	11.6 ± 7.7	1 kHz	16.7 ± 10.2
	2 kHz	11.0 ± 8.0	2 kHz	24.0 ± 18.7
	4 kHz	14.0 ± 8.1	4 kHz	36.2 ± 19.6
	6 kHz	8.4 ± 9.6	6 kHz	41.1 ± 17.8
	8 kHz	12.1 ± 8.5	8 kHz	47.1 ± 18.7

Pure tone audiogram information at each frequency is averaged across both ears and all subjects. BDI, Beck Depression Inventory; BAI, Beck Anxiety Inventory.

### Audiometric evaluation

A comprehensive audiometric evaluation was performed for each subject. Audiological testing took place inside a sound-attenuating booth and included pure tone testing, word recognition testing, and bone conduction testing. Additional tests included distortion product otoacoustic emissions and tympanometry measurements to eliminate any confounding peripheral hearing pathologies. For pure tone testing, the test frequencies included 0.25, 0.5, 1, 2, 4, 6, and 8 kHz. For all of the test frequencies, each subject in the NH group had pure-tone thresholds of 25dB HL or lower. Participants in the HL group had a pure-tone threshold of 30 dB HL or lower for the test frequencies 0.25–2 kHz, with the exception of two HL subjects who had a slightly elevated threshold of 35 dB HL at 1 kHz. For the test frequencies 4–8 kHz, the HL subjects had pure-tone thresholds between 30 and 70 dB HL, ranging from mild to moderately-severe HL. Table 1 contains information about the average hearing at testing frequencies for each subject group. None of the HL participants relied on aided hearing.

### Data acquisition

A 3Tesla Siemens Magnetom Allegra head-only scanner was used to acquire all MRI images. A series of two anatomical and two functional images were acquired- the first functional scan was of the emotional task and the second acquired data on resting state; order of acquisition varied across the subjects. Thirty-two low-resolution T2-weighted structural transversal slices (*TR* = 7260 ms, *TE* = 98 ms) were collected for each volume with a 4.0 mm slice thickness and a 0.9 × 0.9 × 4.0 mm^3^ voxel size [matrix size (per slice), 256 × 256; flip angle, 150°]. We obtained 160 high resolution magnetization-prepared rapid-acquisition with gradient echo (MPRAGE) sagittal slices for each volume that were 1.2 mm in thickness with a 1.0 × 1.0 × 1.2 mm^3^ voxel size [*TR* = 2300 ms; *TE* = 2.83 ms; matrix size (per slice), 256 × 256; flip angle, 9°]. The functional images were acquired using the following parameters: slice thickness, 4 mm; inter-slice gap, 0.4 mm; 32 axial or transverse slices, distance factor, 10%; voxel size, 3.4 × 3.4 × 4.0 mm^3^; field of view (FoV) read, 220 mm; TR, 9000 ms with 2000 ms acquisition time; TE, 30 ms; matrix size (per slice), 64 × 64; flip angle, 90°. Functional images were acquired separately for (a) an emotional task and (b) a resting state study.

#### (a) Emotion task

To mitigate the loud noise of the radio frequency gradients generated during image acquisition from interfering with the perception of the stimuli, we used clustered echo-planar imaging (EPI) (Hall et al., 1999; Gaab et al., 2003; Zaehle et al., 2004). Clustered EPI, or sparse sampling, was chosen particularly to improve the listening environment for the subjects with HL. To reduce scanner noise interference with sound perception, we collected one image volume (2 s acquisition time) post stimulus presentation rather than using continuous image acquisition during a period of “relative quiet” when the radio-frequency gradients were switched off and the only source of ambient noise was the scanner pump. The repetition time was 9 s, and within each trial a 6 s stimulus was presented during a 7 s interval of reduced scanner noise. To optimize the scanning procedure to detect neural response from regions within the limbic system, prior to data acquisition, a custom MATLAB (http://www.mathworks.com/products/matlab/) toolbox was used in order to fine-tune the timing of stimulus presentation relative to image acquisition (Dolcos and McCarthy, 2006). Stimuli were selected from the International Affective Digital Sounds (IADS) database and had normative scores for valence and arousal; sounds were rated on a scale of 1–9 (9 very pleasant, 1 very unpleasant for valence and 9 very arousing, 1 not at all arousing for arousal) (Bradley and Lang, 2007). Normative scores were as follows: pleasant (valence: 6.83 ± 0.54, arousal: 6.46 ± 0.56), unpleasant (valence: 2.78 ± 0.58, arousal: 6.9 ± 0.31) and neutral (valence: 4.81 ±0.43, arousal: 4.85 ±0.57). The normative valence ratings for P and U sounds are statistically different at *p* < 0.00001, but did not differ in arousal scores. Supplementary Table 1 contains a complete list of the affective sounds used in the present study. We presented the sounds in the scanner at a maximum comfort level, as indicated by each participant, during the relatively quiet intervals of image acquisition. Prior to data collection, subjects were given both written and verbal instructions. Additionally, subjects were trained using sounds from the IADS database, different from the stimuli chosen in the experiment, to familiarize the participants with the task. During the final experiment, Presentation 14.7 software (www.neurobs.com) on a Windows XP computer in the fMRI control room was used to deliver sounds and instructions via pneumonic headphones (Resonance Technology, Inc., Northridge, CA.). To complete the task, subjects listened to 90 affective sounds [30 pleasant (P), 30 neutral (N), 30 unpleasant (U)], each 6 s in duration and were instructed to rate the sound as P, N, or U as soon as they felt confident in their rating.

#### (b) Resting state

To acquire information about the resting state, continuous scanning instead of clustered image acquisition was employed. During the resting scan, which was continuous and lasted approximately 5 min, subjects were instructed to lie still and to look at a fixation cross for the scan duration. One hundred and fifty volumes were collected for each subject. The first four images were discarded prior to preprocessing, leaving 146 volumes for analysis.

### Preprocessing

Pre-processing was similar for both types of functional scans. Statistical Parametric Mapping 8 (SPM, Welcome Trust Centre for Neuroimaging, http://www.fil.ion.ucl.ac.uk/spm/software/spm8/) software was used to analyze the functional imaging data. The images were first realigned using a rigid body transformation to control for head motion. Next, the low resolution Axial T2 (AxT2) image was registered to the mean fMRI image generated during the first step. The high resolution MPRAGE image was then registered to the AxT2 image. To normalize the functional images to MNI space, the MPRAGE was normalized to match a standard T1 MNI template. The normalized images were then smoothed using a Gaussian kernel of 8 × 8 × 8 mm^3^ full width at half-maximum. To account for artifacts created by head motion, data from 3 NH subjects who displayed excessive motion (defined as at or above ±1.5 mm translation and ± 1.5° rotation in any direction) were not included for further analysis. We also included regressors of motion as covariates of no-interest in the general linear models created in the different statistical analyses, in order to (partially) remove motion-related artifacts. Further, *t*-tests of root-mean-square estimates of both rotational and translational movement showed that there was no statistical significant difference between the two groups (translational motion mean ± standard deviation): NH (0.63 ± 0.28); HL (0.67 ± 0.23); rotational motion: NH (0.01 ± 0.005), HL (0.01 ± 0.004).

### Data analysis

#### (a) Emotion task

Behavioral data were obtained in the scanner during fMRI data acquisition. We collected individual subject ratings of each sound as P, N, or U along with reaction times. Subject ratings and reaction times were analyzed using separate Two-Way ANOVA tests in SPSS ver. 20 software (statistical package for social sciences, IBM, http://www-01.ibm.com/software/analytics/spss/). Group (NH, HL) and condition (P, N, U) were set as independent fixed factors in a general linear model within SPSS, and significance was set at *p* < 0.05.

For data analysis, trials were coded based upon each individual's subjective rating of the affective sounds. We chose to employ the individual ratings to classify trials as “P,” “N,” or “U,” rather than the norms reported in IADS. Either using the IADS classification or individual rating to analyze data are valid methods of classifying the individual trials for further analysis. The trend in affective neuroscience literature is to move away from the normative classification to the individual classification, particularly when examining special populations where it is expected that emotional responses are altered (e.g., older adults or patient population) (St Jacques et al., 2010). It should be noted that the ratings reported with the IADS were obtained from younger adults with NH (Bradley and Lang, 2007). In the present study, the subject population was older with mean age of 51.4 ± 9.9 for NH adults and 58.2 ± 9.5 for those with HL. Therefore, individual ratings were used rather than the norms for classifying the trials obtained during fMRI scanning. First level fixed effects analysis was performed on each subject's smoothed images to generate P > N and U > N contrast images. Motion parameters were included in the first level model as covariates of no-interest. The contrast images were then included in the flexible factorial analysis and *post-hoc* two-sample *t*-tests at the second level. The P + U > N contrast was computed by performing a *t*-test on the condition vectors for each group separately in the flexible factorial model. The three factor design included group, subject and condition. Group was assumed to be independent and have unequal variance, subject was assumed to be independent and possess equal variance, and condition was assumed to be a dependent factor and to have equal variance. To directly compare the NH and HL groups, we conducted *post-hoc* two-sample *t*-tests within the flexible factorial model. Additionally, we performed a region-of-interest (ROI) analysis using the Wake Forest University (WFU) pickatlas toolbox (http://www.fmri.wfubmc.edu) within SPM8, with regions defined anatomically based on the human MNI atlas within the toolbox. Based on our *a priori* hypothesis about the involvement of auditory and limbic regions in affective sound processing, we created a single anatomically-defined mask via selecting regions including the amygdala, insula, parahippocampus, nucleus accumbens, ventral medial prefrontal cortex, inferior colliculus, medial geniculate body and primary auditory cortex (Brodmann areas 42, 41, 22). ROI analysis was performed on the NH (P + U > N), HL (P + U > N), and between group contrasts, and small volume correction (SVC) was employed. All clusters identified in the results were reported using a significance level set at *p* < 0.025 FWE at either the voxel or cluster level (threshold halved from the standard *p* < 0.05 to account for a two-tailed *t*-test).

#### (b) Resting state

Preprocessing of the resting state data began with slice time correction for the interleaved, ascending data collection. Following that, the same preprocessing steps used for the emotion task were applied. The Functional Connectivity Toolbox (Conn) (Whitfield-Gabrieli and Nieto-Castanon, 2012) for MATLAB was used for data analysis. The smoothed fMRI data was band-pass filtered from 0.008 to 0.08 kHz. The average BOLD timeseries of the segmented white matter and cerebrospinal fluid as well as the realignment/motion parameters generated during preprocessing were regressed out of the data. Then, seed-to-voxel analysis was performed to analyze the auditory resting state network, the DMN, and the DAN. Connectivity was assessed using pairs of seed regions for both the DMN and auditory networks; correlations between each seed and the whole brain were measured and averaged across seed pairs. Seed locations are listed in Table 2. For the auditory network, seeds were located in the bilateral primary auditory cortices. For the DMN, they were located in the medial prefrontal cortex and the posterior cingulate cortex. The DAN was examined using four seeds in the left and right posterior intraparietal sulci and the left and right frontal eye fields (Burton et al., 2012). All seeds were created using Marsbar (Brett et al., 2002) and were 5 mm in radius. Coordinates of seed regions were the same as those used in (Schmidt et al., 2013). The resting state data used in the present study were partially described in the Schmidt et al. (2013) study, but have been re-analyzed for the purpose of this study. Correlation maps of the whole brain were created for each seed and then averaged over all seeds for a specific network for each subject. These correlations were then z-transformed, group averages were computed, and across-group comparisons were made via two-sample *t*-tests in the Conn toolbox (Whitfield-Gabrieli and Nieto-Castanon, 2012). Results were then exported to SPM8 for display purposes. After whole brain analysis at *p* < 0.001 uncorrected threshold, clusters that were significant at *p* < 0.025 FWE either at voxel or cluster level were selected to account for both tails of the *t*-test, with cluster extent set at 25 voxels.

**Table 2:** **Seed regions for the resting state functional connectivity analysis, consisting of seeds for canonical resting state networks and for networks based on local axima from the results of the emotion task study**.

**Network**	**Seed region**	**MNI coordinates *X, Y, Z***
Auditory	Left primary auditory cortex	55, −22, 9
Auditory	Right primary auditory cortex	−41, −27, 6
DMN	Medial prefrontal cortex	8, 59, 19
DMN	Posterior cingulate cortex	−2, −50, 25
DAN	Left posterior intraparietal sulcus	−23, −70, 46
DAN	Right posterior intraparietal sulcus	26, −62, 53
DAN	Left frontal eye field	−25, −11, 54
DAN	Right frontal eye field	27, −11, 54
	Left amygdala	−30, −2, −18
	Left inferior parietal lobule	−44, −36, 26
	Left superior frontal gyrus	−24, 42, 30
	Right middle temporal gyrus	44, −62, 22
	Right superior parietal lobule	30, −62, 44

Coordinates are listed in Montreal Neurological Institute (MNI) coordinates.

A seeding analysis designed to examine resting state network connectivity was performed using ROIs determined from published studies as stated earlier, as well as using ROIs identified from the task-based study. The latter ROIs included the left amygdala, left inferior parietal lobule, left superior frontal gyrus, right middle temporal gyrus, and the right superior parietal lobule (Table 2). The left amygdala seed was created based on the NH > HL (P + U > N) contrast from the task ROI analysis. The right superior parietal lobule and left inferior parietal lobule were both based on the HL > NH (P + U > N) contrast, and the right middle temporal gyrus and left superior frontal gyrus seeds were based on the NH > HL (P + U > N) contrast from the emotion task results. All of these ROIs were determined from group-level contrasts. Peak maxima were used as the centers for the spherical ROIs, and the mean BOLD timeseries of the voxels in the ROI was generated. Seed specification, data generation and statistical analyses were performed in the manner described earlier for the standard seeds.

## Results

### Behavioral data

#### (a) Emotion task

**Figure 1:**
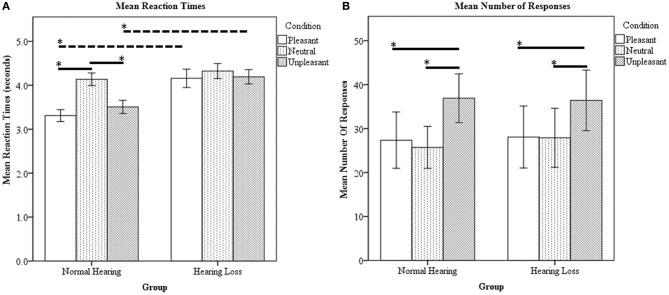
**Affective sound categorization task behavioral results. (A)** Mean reaction time data. For within group comparison, the HL group did not statistically differ between the P, N, and U reaction times. However, the NH group responded significantly slower to the N sounds compared to the P and U stimuli. Compared to the NH group, the HL reaction times were significantly slower for P and U sounds. **(B)** Mean number of responses. The NH and HL groups responded U significantly more than N and P. Statistical significance level *p* < 0.05 indicated by ^*^.

Ratings were obtained in the scanner simultaneous with fMRI data acquisition. There was a statistically significant main effect of group [*F*_(1, 23)_ = 69.53, *p* < 0.000001], main effect of condition [*F*_(1, 23)_ = 17.59, *p* < 0.000001] and interaction between group and condition [*F*_(1, 23)_ = 7.79, *p* < 0.000423] for the reaction time data. The NH group responded significantly slower to the neutral sounds compared to the pleasant and unpleasant sounds (Figure 1A). The HL group's reaction time for the three types of sounds did not significantly differ (Figure 1A). For between group comparisons, the HL group was significantly slower for both pleasant and unpleasant sounds compared to the NH group (Figure 1A). Concerning the type of responses, there was a significant main effect of condition [*F*_(1, 23)_ = 7.162, *p* < 0.01]; however, the main effect of group [*F*_(1, 23)_ = 0.118, *p* = 0.733] and the interactions [*F*_(1, 23)_ = 0.109, *p* = 0.897] did not reach significance. Both groups rated significantly more stimuli as unpleasant compared to pleasant and neutral (determined using *post-hoc* within-group *t*-tests) (Figure 1B). Note that the experimental design used an equal number of sounds classified as P, N, and U according to the normative IADS scores (Bradley and Lang, 2007). Due to the observed variation from the normative ratings, we chose to code the trials during analysis according to each individual's rating.

#### (b) Resting state

No behavioral data were obtained for the resting state study.

### fMRI data

#### (a) Emotion task

**Figure 2:**
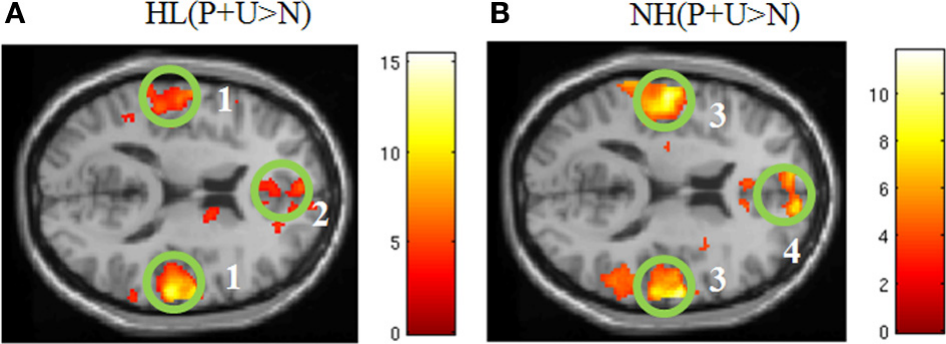
**Statistical parametric maps for the effect of affective stimuli (P + U > N) for each group separately (A,B). (A)** HL (P + U > N) and **(B)** NH (P + U > N) images illustrate the similar whole brain response patterns from both groups (MNI coordinate *z* = +14). The maps are displayed at *p* < 0.001 uncorrected level for better visualization, but the clusters in the circles are corrected for multiple comparisons (*p* < 0.05 FWE). (1) bilateral middle temporal gyrus, (2) medial frontal gyrus, (3) bilateral middle temporal gyrus, (4) medial frontal gyrus.

Within group comparisons: In the NH group, areas of increased activation for the contrast P + U > N were observed in the bilateral middle temporal gyri, right transverse temporal gyrus, left superior temporal gyrus, left post central gyrus, right medial frontal gyrus, left superior frontal gyrus, left middle frontal gyrus, left anterior cingulate and the left insula (Figure 2, Table 3). With respect to the HL group, increased response was obtained in the bilateral superior temporal gyri, bilateral transverse temporal gyri, right middle temporal gyrus, right superior frontal gyrus, left middle frontal gyrus, right medial frontal gyrus, right precuneus, left inferior parietal lobule, left precentral gyrus, left lentiform nucleus and corpus callosum for affective sounds compared to neutral sounds (Figure 2, Table 3). ROI analysis revealed increased response in bilateral transverse temporal gyri, bilateral superior temporal gyri, bilateral medial frontal gyrus, left insula, right middle temporal gyrus and right parahippocampus for the NH (P + U > N) contrast (Table 3). For the HL (P + U > N) comparison, increased response was observed in the bilateral transverse temporal gyri, bilateral superior temporal gyri, bilateral superior frontal gyri, right medial frontal gyrus and left insula (Table 3).

**Table 3:** **Local maxima for the P + U > N contrasts from the emotion task**.

**Contrast**	**MNI coordinates *X, Y, Z***	***Z*-score**	**Cluster (voxels)**	**Gyrus (brodmann area)**
NH group (P + U > N)	62, −10, 12	6.45	3293	R. transverse temporal gyrus (BA 42)
	58, 2, −8	6.42		R. middle temporal gyrus (BA 21)
	56, 6, −18	5.98		R. middle temporal gyrus (BA 21)
	−58, −16, 15	6.06	3974	L. post central gyrus (BA 40)
	−54, −6, 0	5.95		L. superior temporal gyrus (BA 22)
	−66, −28, 4	5.73		L. superior temporal gyrus (BA 22)
	−24, 66, 14	5.59	218	L. superior frontal gyrus (BA 10)
	−32, 62, 16	5.48		L. middle frontal gyrus
	−38, −58, 2	5.57	233	L. middle temporal gyrus (BA 37)
	−40, −34, 20	5.39	10	L. insula (BA 13)
	4, 56, 20	5.38	1765	R. medial frontal gyrus (BA 9)
	10, 60, 16	5.32		R. medial frontal gyrus (BA 10)
	−8, 34, 6	5.12		L. anterior cingulate (BA 32)
HL group (P + U > N)	58, −14, 12	7.21	2289	R. transverse temporal gyrus (BA42)
	60, −22, 14	6.46		R. superior temporal gyrus (BA 42)
	62, −28, 4	5.30		R. superior temporal gyrus (BA 22)
	−42, −36, 22	6.56	2471	L. inferior parietal lobule (BA40)
	−50, −6, 6	5.72		L. precentral gyrus (BA 6)
	−56, −16, 6	5.29		L. superior temporal gyrus (BA 22)
	−62, −10, 12	5.13		L. transverse temporal gyrus (BA42)
	−46, 36, 28	5.64	760	L. middle frontal gyrus (BA46)
	−24, −16, −4	5.64	195	L. lentiform nucleus
	44, −62, 24	5.52	405	R. middle temporal gyrus (BA 39)
	36, −74, 18	5.14		R. middle temporal gyrus (BA 39)
	−8, 32, 6	5.48	1644	Corpus Callosum
	8, 42, −10			R. medial frontal gyrus (BA10)
	24, −50, 52	5.25	63	R. precuneus (BA 7)
	12, 62, 28	5.10	392	R. superior frontal gyrus (BA10)
	−30, 46, 40	5.07	43	L. middle frontal gyrus (BA9)
[ROI] NH group (P + U > N)	62, −10, 12	6.45	534	R. transverse temporal gyrus (BA 42)
	58, 0, −6	6.16		R. superior temporal gyrus (BA 22)
	54, −24, 6	4.90		R. superior temporal gyrus (BA 41)
	52, −34, 2	4.45		R. middle temporal gyrus (BA 22)
	−58, −16, 12	5.94	938	L. transverse temporal gyrus (BA 42)
	−66, −28, 4	5.73		L. superior temporal gyrus (BA 22)
	−50, −22, 16	5.68		L. insula
	−40, −34, 20	5.39		L. insula (BA 13)
	−48, −26, 18	5.38		L. insula (BA 13)
	−54, −20, 6	5.25		L. superior temporal gyrus (BA 41)
	−52, −26, 12	4.69		L. transverse temporal gyrus (BA 41)
	6, 58, 20	5.30	54	R. medial frontal gyrus (BA 10)
	−6, 58, 14	4.52	41	L. medial frontal gyrus (BA 10)
	20, −20, −22	4.31	42	R. parahippocampus (BA 28)
[ROI] HL group (P + U > N)	60, −12, 12	6.72	510	R. transverse temporal gyrus (BA42)
	60, −22, 12	6.18		R. superior temporal gyrus (BA 42)
	56, −10, 8	5.96		R. superior temporal gyrus (BA 22)
	52, −22, 14	5.11		R. insula
	−52, −6, 4	5.60	486	L. superior temporal gyrus (BA 22)
	−42, −34, 20	5.60		L. insula (BA 13)
	−62, −10, 12	5.08		L. transverse temporal gyrus (BA 42)
	−54, −28, 10	4.71		L. superior temporal gyrus (BA 41)
	8, 42, −10	5.31	201	R. medial frontal (BA 10)
	12, 62, 28	5.10	13	R. superior frontal gyrus (BA 10)
	−14, 62, 26	4.76	15	L. superior frontal gyrus (BA 10)

The loci are listed for each group separately for the whole-brain analysis and the region-of-interest (ROI) analysis. Reported regions are listed in Montreal Neurological Institute (MNI) coordinates and in terms of Brodmann areas (before determining the Brodmann areas the MNI coordinates were converted to Talairach coordinates). The ROI mask comprised of bilateral regions in the primary auditory cortex, medial geniculate body, inferior colliculus, amygdala, insula, parrahippocampus, nucleus accumbens, and ventral medial prefrontal cortex, defined anatomically. Statistical threshold was set at p < 0.05 FWE corrected for multiple comparisons; all clusters noted here were significant at both voxel and cluster level. L, left; R, right.

**Figure 3:**
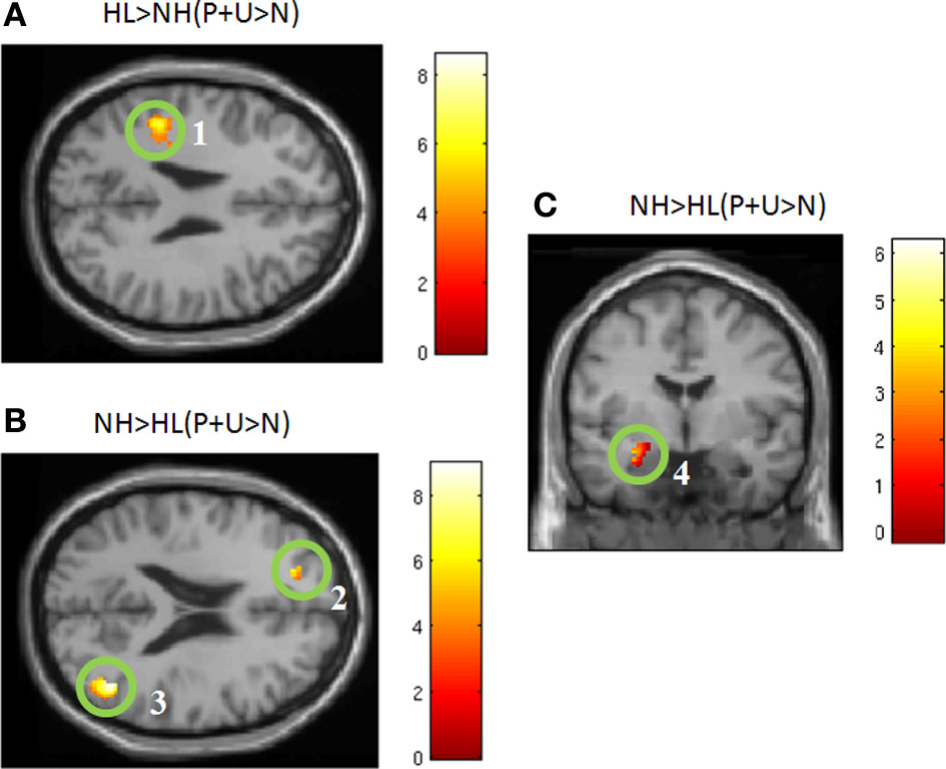
**Statistical parametric maps for *post-hoc* two-tailed two sample *t*-tests and region-of-interest analysis (ROI)**. **(A)** HL > NH (P + U > N) and **(B)** NH > HL (P + U > N) illustrate the brain regions chosen from the *post-hoc* two sample *t*-tests for the seed analysis (MNI coordinate *z* = +26, *z* = +21, respectively). **(C)** Denotes increased amygdala activation observed for the NH > HL (P + U > N) comparison (MNI coordinate y = −4). The maps are displayed at *p* < 0.001 uncorrected level for better visualization, but the clusters in the circles are corrected for multiple comparisons (*p* < 0.025 FWE). (1) left inferior parietal lobule, (2) left superior frontal gyrus, (3) right middle temporal gyrus, (4) left amygdala.

Between-group comparisons: For the NH > HL (P + U > N) comparison, we observed heightened response in the left superior frontal gyrus, right middle temporal gyrus, left superior temporal gyrus, and left superior occipital gyrus (Figure 3, Table 4). Concerning the HL > NH (P + U > N) comparison, elevated response was observed in the right superior parietal lobule, right precuneus, and left inferior parietal lobule (Figure 3, Table 4). For the ROI analysis, no suprathreshold voxels were obtained for the HL > NH (P + U > N) contrast. However, for the NH > HL (P + U > N) comparison, increased response was observed in the left amygdala and left parahippocampus (Figure 3, Table 4).

**Table 4:** **Local maxima for the P + U > N contrasts from the emotion task for the group comparisons**.

**Contrast**	**MNI coordinates *X, Y, Z***	***Z*-score**	**Cluster (voxels)**	**Gyrus (brodmann area)**
NH > HL (P + U > N)	44, −62, 22	5.77	261	R. middle temporal gyrus (BA 39)
	−34, 2, −18	5.72	133	L. superior temporal gyrus (BA 38)
	−24, 42, 20	5.04	319	L. superior frontal gyrus (BA 10)
	−30, −78, 24	4.45^*^	272	L. superior occipital gyrus (BA 19)
HL > NH (P + U > N)	18, −64, 44	5.62	402	R. precuneus (BA 7)
	30, −62, 44	4.86		R. superior parietal lobule (BA 7)
	−44, −36, 26	4.68^*^	288	L. inferior parietal lobule (BA 40)
[ROI] NH > HL (P + U > N)	−30, −2, −18	4.80	57	L. amygdala/parrahippocampus
	−34, 2, −22	4.62		L. parrahippocampus
[ROI] HL > NH (P + U > N)				No significant regions

The loci are listed for each group separately for the whole-brain analysis and the region-of-interest (ROI) analysis. Reported regions are listed in Montreal Neurological Institute (MNI) coordinates and in terms of Brodmann areas (before determining the Brodmann areas the MNI coordinates were converted to Talairach coordinates). The ROI mask comprised of bilateral regions in the primary auditory cortex, medial geniculate body, inferior colliculus, amygdala, insula, parrahippocampus, nucleus accumbens, and ventral medial prefrontal cortex, defined anatomically. Statistical threshold was set at p < 0.025 FWE corrected for multiple comparisons (to account for two-tailed t-test); all clusters noted here were significant at both voxel and cluster level, unless noted with an

* which indicates significance only at cluster level. L, left; R, right.

#### (b) Resting state

**Figure 4:**
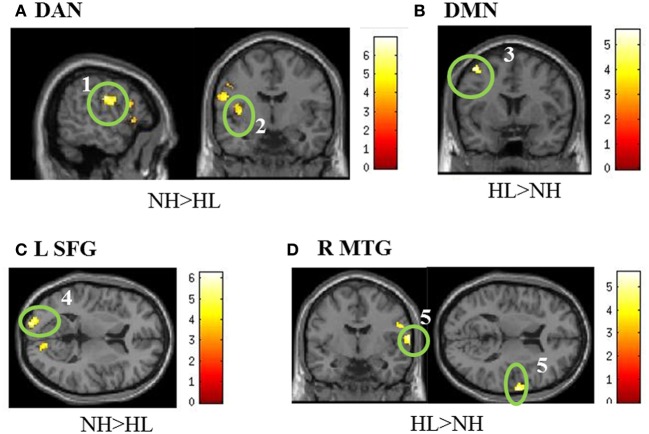
**Statistical parametric maps of the two-tailed two sample *t*-tests of the resting state functional connectivity results. (A,B)** show the results of the seeding analyses designed to examine the connectivity of the resting state networks, while **(C,D)** show correlations to the task-based seeds. The maps are displayed at *p* < 0.001 uncorrected level for better visualization, but the clusters in the circles are corrected for multiple comparisons (*p* < 0.025 FWE). In **(A)**, the seed regions were located in the bilateral posterior intraparietal sulci and the bilateral frontal eye fields to examine connectivity in the dorsal attention network (DAN). For the default mode network (DMN) in **(B)**, seeds were located in the posterior cingulate and medial prefrontal cortices. **(C,D)** are labeled with the seed regions in the figure. (1) left postcentral gyrus, (2) left insula, (3) left middle frontal/precentral gyrus, (4) left middle occipital, (5). right precentral gyrus. MNI coordinates for the different sub- figures are: **(A)** *x* = −56, *y* = 6, **(B)** *y* = 6, **(C)** *z* = −18 left, **(D)** y = −6, *z* = 12. Abbreviations: DAN, dorsal attention network; DMN, default mode network; L SFG, left superior frontal gyrus; R MTG, right middle temporal gyrus.

No significant differences were found between groups in the auditory resting state network. With respect to the DMN, a significant difference in the left middle frontal/precentral gyrus was observed in the HL > NH comparison. Analysis of the DAN revealed a significant difference in the left postcentral/precentral gyrus and left insula. These results of the typical intrinsic networks are listed in Table 5 and displayed in Figure 4. For the connectivity analysis with task-based ROIs, no significant differences in connectivity with the left amygdala were seen. Placing a seed in the left inferior parietal lobule also did not reveal significant differences. With the seed in the left superior frontal gyrus, the NH > HL contrast showed significant differences in connectivity with the left middle occipital lobe. The right middle temporal seed showed a stronger correlation with the right precentral gyrus in the HL group compared to the NH group. Finally, no connectivity differences were seen with the seed in the right superior parietal lobule. The results of this analysis are also shown in Table 5 and Figure 4.

**Table 5:** **Local maxima for results of the resting state functional connectivity analysis**.

**Network/seed**	**Contrast**	**MNI coordinates *X, Y, Z***	***Z*-score**	**Cluster (voxels)**	**Gyrus (brodmann area)**
AUD	NH > HL				No significant regions
	HL > NH				No significant regions
DMN	NH > HL				No significant regions
	HL > NH	−44, 6, 58	4.38	107	L. middle frontal/precentral gyrus (BA 6)
DAN	NH > HL	−56, −12, 26	5.00	171	L. postcentral gyrus
		−56, −2, 26	4.09		L. precentral gyrus
		−52, −4, 38	3.36		L. postcentral gyrus
		−38, −6, 6	4.36	118	L. insula
		−40, −2, 16	4.00		L. precentral gyrus (BA 6)
		−46, −10, 14	3.43		L. precentral gyrus (BA 6)
	HL > NH				No significant regions
l amyg	NH > HL				No significant regions
	HL > NH				No significant regions
l inf pariet	NH > HL				No significant regions
	HL > NH				No significant regions
l sup front	NH > HL	−18, −96, 8	4.7	167	L. middle occipital gyrus (BA 18)
		−4, −94, 20	4.38		L. cuneus (BA 19)
	HL > NH				No significant regions
r mid temp	NH > HL				No significant regions
	HL > NH	66, −6, 12	4.39	114	R. precentral gyrus (BA 22)
r sup pariet	NH > HL				No significant regions
	HL > NH				No significant regions

The seeds are listed for the canonical resting state networks first, followed by seeds from the task-based study. Reported regions are listed in Montreal Neurological Institute (MNI) coordinates and in terms of Brodmann areas (before determining the Brodmann areas the MNI coordinates were converted to Talairach coordinates). Statistical threshold was set at p < 0.025 FWE corrected for multiple comparisons (to account for two-tailed t-test). All regions were significant at the cluster level. Abbreviations: L, left; R, right; amyg, amygdala; inf pariet, inferior parietal; sup front, superior frontal; mid temp, middle temporal; sup pariet; superior parietal.

## Discussion

We used a combined task- and rest-based fMRI study to identify the influence of HL on auditory and emotion processing. Results of the responses collected in the scanner revealed similar ratings for the unpleasant, pleasant, and neutral sounds—both groups tended to rate more sounds as unpleasant relative to the other types of sounds. However, the HL group differed from the NH group in their response times, which were significantly slower for the affective sounds. Overlapping patterns of fMRI activation were observed in both groups when processing affective sounds compared to neutral sounds. The main finding for the emotion task was increased activation in the left amygdala/parahippocampal gyrus complex for the NH > HL (P + U > N) comparison, via targeted ROI analysis. The reverse contrast, HL > NH (P + U > N), did not show increased activation within the limbic system, but rather revealed heightened responses in the right superior parietal lobule, right precuneus, and left inferior parietal lobule. Resting-state functional connectivity in the same group of participants focused on the canonical intrinsic networks and on seed regions obtained from the task-based activation patterns. Among the typical intrinsic networks, the default mode and DAN, but not the auditory network, revealed differences between the groups. Using seeds from the local maxima noted in the task-based analysis, decreased connectivity between the frontal cortex and other brain regions was noted, with the exception of stronger connectivity between the middle temporal gyrus and the right precentral gyrus in the HL group compared to the NH group. Our results suggest that HL may alter the emotional processing networks and lead to slower reaction times to affective stimuli.

### Emotion task

HL may reduce the engagement of the emotional processing system, either because of disordered processing of acoustic or of valence features. Complex anatomical and functional connections exist between the auditory cortex and the limbic system, primarily with the amygdala (Amaral and Price, 1984; Blood and Zatorre, 2001; Koelsch et al., 2006; Tschacher et al., 2010; Kumar et al., 2012). Forward projections from the auditory cortex to the amygdala have been shown to be modulated by acoustic features, but backward projections appear to be modulated by the valence of sound (Kumar et al., 2012). The forward and backward projections work in concert to interpret incoming affective stimuli (Kumar et al., 2012). Sound deprivation may reduce the amount of acoustic or valence information available for this network. Reduced information may result in a dampened emotional response to affective stimuli, because individuals with HL may not receive all of the acoustic or valence information necessary to cause a robust emotional response. We investigated this hypothesis via whole brain analysis and a targeted ROI analysis of the auditory and limbic areas.

In our study, both positively and negatively valent sounds exhibited greater engagement of the limbic system and faster response times, compared to neutral sounds, in NH individuals. However, this pattern differed in the HL group, with a decreased response in the temporal and frontal cortices (whole brain analysis) and in the amygdala and parahippocampus (ROI analysis), and an increased response in the parietal cortices and precuneus compared to the NH group when processing affective sounds (Table 4). Similarly, in behavioral responses, the reaction times for the P and U sounds were significantly slower in the HL group (Figure 1A). The behavioral data suggest that the advantageous faster processing of affective sound found in the NH group does not occur in the HL group. The slower responses to pleasant and unpleasant sounds in the HL group may be due to lack of faster, bottom-up engagement of the amygdala and other limbic structures during auditory processing. However, HL does not appear to affect the response to all sounds, with the reaction times for neutral sounds being nearly identical in the two groups. Instead, the highly valent sounds were most affected, indicating that it is identification of valence information rather than acoustic information that is impacted by HL. Another possible explanation of the differential processing of affective sounds by the HL group could be that there is more energy or information in the high frequency regions in the affective sounds and less so in the neutral sounds. Difficulty of processing high-frequency information in the affective sounds by the HL group led to longer reaction times. In order to maintain ecological validity, we chose not to low-pass filter the sounds to compensate for the HL of the HL group. In a previous study with mild-to-moderate HL (Husain et al., 2011b), we filtered sounds such that there was no energy in frequencies greater than 2 kHz. We found no difference in accuracy or reaction times for a discrimination task between HL and NH groups, nevertheless, the fMRI activation patterns were different (Husain et al., 2011b). In sum, regardless of the actual mechanism, our results suggest that HL may reduce engagement of the amygdala and result in slower reaction to positively and negatively valent sounds.

Another interesting aspect of the behavioral results was the finding that an increased number of sounds were classified as unpleasant, which differed from the published normative data (Bradley and Lang, 2007). There are at least two possible explanations for this finding. First, the normative scores were obtained in a young, NH population; therefore it is not surprising that both sets of older middle-aged participants in our study differed from these ratings, which points to an effect of age. Another possible explanation is that possible discomfort in the scanner may have influenced our participants to be more negative in their ratings. In order to tease apart these explanations and better understand the effect of aging on emotional processing, we intend to conduct a follow-up study with both young and older participants using stimuli from the IADS database.

### Resting state data

Resting state functional connectivity demonstrated alterations in the frontal cortex in HL patients. Increased connectivity between frontal regions and seed regions for the DAN and DMN was seen in HL patients compared to NH controls. A decreased correlation was seen between the left superior frontal cortex and the left middle occipital gyrus in HL subjects compared to controls. The relationship seen between HL and alterations in frontal connectivity may not just be important at baseline, as engagement of frontal regions was also apparent during the emotion task. The cause of these network alterations is not clear. The alterations could be purely attentional in nature, but they may also be a factor of interactions between emotional and attentional systems. A study including an attentional task without an emotional component may help to clarify this.

Except for activation of the left temporal pole, we did not find evidence to support our expectation that the response of the auditory cortex would differ between the two groups when processing affective sounds. A separate resting-state functional connectivity analysis with seeds in the primary auditory cortices also failed to find significant connectivity differences at rest between the two groups. The lack of significant findings may relate to mild-to-moderate severity of the HL in our study. To date, there have been no studies of resting state functional connectivity in patients with this level of HL. Deafness, however, has been investigated in this context. Intrinsic connectivity has been shown to be impacted by deafness, both within and outside of the temporal cortex (Li et al., 2013). The findings of the (Li et al., 2013) study are similar to those in our own study. Deaf patients showed increased negative correlations between the middle superior temporal sulcus and the left middle occipital and right precentral gyri when compared to NH controls. In our study, the left middle occipital gyrus was shown to be less correlated to the left superior frontal gyrus in the HL group, whereas the right precentral gyrus was more correlated with the right middle temporal gyrus. The presence of altered connectivity in similar regions in both deaf and our mild-to-moderate HL patients warrants further resting state connectivity studies examining varying degrees of HL.

It is important to note that inferences about the directionality of the connections cannot be made from the present functional connectivity analysis. An effective connectivity analysis, perhaps implemented as a structural equation modeling or dynamic causal modeling, would be needed to examine directionality (Horwitz, 2003). Our results suggest only a general alteration in connectivity between two related regions; differences in correlation may not be specifically due to coupling between the seed and a particular region, but may also arise due to the influence of a third region, or changes in noise, etc. (Friston, 1994).

HL is positively correlated with age (Yueh et al., 2003); age is therefore a potential confound in our research. A study examining connectivity in resting state networks (Onoda et al., 2012) noted a significantly decreased correlation between the auditory resting state network and the salience network (which includes the insula, ventrolateral prefrontal cortex (VLPFC), thalamus and cerebellum) with age. In addition, connections between regions of the salience network also weakened with age. The regions of the salience network relate to the processing of emotional stimuli. It is possible that HL within the older population in this study is partially responsible for the observed results. The decreased correlation between regions of the DAN and the insula seen in our study fit well with the results of the aging study. In our own study, however, all participants were matched for age; therefore, we are unable to parse out the effects of age from those of HL. More fMRI studies specifically addressing the effects of HL of varying severity, in different age groups, should be performed to clarify its impact on intrinsic and task-based functional networks. Subject motion in the scanner is another notable confound with the resting state data being particularly sensitive to its influence. Although we excluded data from participants who exhibited excessive head motion, included motion parameters as covariates of no-interest in our statistical models and statistical tests did not show any significant differences between the groups, it is possible that motion-related artifacts continue to affect our results, as shown by recent publications (Kundu et al., 2012; Van Dijk et al., 2012; Kundu et al., 2013; Power et al., 2014). Future studies with more stringent data acquisition considerations and more advanced analytical methods will need to be conducted to fully account for the possibility of motion artifacts.

## Conclusion

Our results suggest that HL may affect emotional processing by decreasing amygdalar recruitment, resulting in slower reaction times to highly valent sounds. Although the HL group demonstrated slower response times to affective sounds, there was no difference in sound ratings between the HL and the NH group. Altered engagement of the frontal regions was also demonstrated in both emotion task-based subtraction and resting state functional connectivity analyses. HL is the third most common chronic condition in older adults and is highly comorbid with another hearing disorder, tinnitus. Our results in those with unaided, mild-to-moderately severe HL have implications for auditory rehabilitation for hearing impairment, reducing social isolation in the elderly, and management strategies for tinnitus.

### Conflict of interest statement

The authors declare that the research was conducted in the absence of any commercial or financial relationships that could be construed as a potential conflict of interest.

## Supplementary material

Supplementary Table 1 **Sounds included in the study**. Separated by column are 30 pleasant, 30 unpleasant, and 30 neutral sounds chosen from the IADS database to be included in the study.
