# Supplementary material for: The effect of mild-to-moderate hearing loss on auditory and emotion processing networks
Source: Front Syst Neurosci. 2014 Feb 4;8:10. doi: 10.3389/fnsys.2014.00010 (PMC3912518; doi:10.3389/fnsys.2014.00010)
Supplement: Supplementary Table 1 — Sounds included in the study. Separated by column are 30 pleasant, 30 unpleasant, and 30 neutral sounds chosen from the IADS database to be included in the study. [file DataSheet1.PDF]

Supplementary Table 1

**Sound Category:**	**Pleasant**	**Unpleasant**	**Neutral**
**Sound Stimulus:**	Video Game	Growl1	Male Snore
	Kids2	Puppy	Hiccup
	Carousel	Baby Cry	Office1
	Music Box	Buzzing	Paper2
	Colonial Music	Gun Shot	Wind
	Funk Music	Vomit	Shovel
	Wedding	Explosion	Clock
	Boy Laugh	Crowd1	Writing
	Baby	Creep	Radio
	Doorbell	Babies Cry	Cat
	Casino1	Crowd3	Pig
	Horse Race	Dentist Drill	Toilet
	Crowd4	Plane Crash	Paper1
	Party	Siren2	Heart Beat
	Bugle	May Day	Rain1
	SlotMachine1	Crash	Walking
	EroticFem3	Sirens	Helicopter2
	Countdown	Rattle Snake	Brush Teeth
	SlotMachine2	Bees	Lawnmower
	Baseball	Attack3	Fan
	Casino2	Car Horns	Panting
	RockNRoll	Air Raid	Type Writer

	Sports Crowd	Fight2	Train
	Erotic Couple	Child Abuse	Rooster
	Crowd2	Male Scream	Night
	EroticFem2	Bike Wreck	Cows
	Bongos	Attack3	Dog
	EroticFem1	Siren1	Country Night
	EroticCouple2	Tire Skids	Chickens
	Roller Coaster	Alarm Clock	Tropical
